# Influence of upper limb activity on the step count and accuracy of sleep time of a wristband-type physical activity tracker

**DOI:** 10.1371/journal.pone.0271155

**Published:** 2022-07-08

**Authors:** Nobuyuki Sano, Takanori Taniguchi, Hisato Nakazono

**Affiliations:** 1 Department of Occupational Therapy, Faculty of Medical Science, Fukuoka International University of Health and Welfare, Fukuoka, Fukuoka, Japan; 2 Department of Physical Therapy, Faculty of Medical Science, Fukuoka International University of Health and Welfare, Fukuoka, Fukuoka, Japan; University of Essex, UNITED KINGDOM

## Abstract

**Background:**

A wristband-type consumer physical activity tracker (PAT) is commonly used in rehabilitation to assess an individual’s physical activity. However, under the free-living setting, the wristband-type PAT tends to overestimate step counts when compared with the research-standard criterion. Also, daily rhythm characteristics, such as sleep time, are difficult to monitor accurately based solely on self-reporting.

**Purpose:**

To identify the conditions measured as step counts by a wristband-type consumer PAT when using the upper limbs in daily living, and the measurement accuracy of the sleeping time estimated from the wristband-type PAT.

**Methods:**

Forty participants (20 females, mean age 32.65 ± 9.52 years) were enrolled in two experiments in this study. In Experiment 1, we measured the influence of upper limbs activity (movement speed and distance) on step counts of wristband-type and waist holder-type PAT in two upper limb tasks. In Experiment 2, we verified the measurement accuracy of two sleep times by wristband-type PAT using a self-reported survey for 3 days.

**Results:**

The results of Experiment 1 revealed that the step counts using wristband-type PAT were influenced by upper limbs activity depending on movement distance (F (1, 19) = 31.705, p < 0.001) but not speed (F (1, 19) = 2.669, p < 0.117). Whereas, there was no relationship between step counts and upper limb activity in waist holder-type PAT. The results of Experiment 2 showed that the sleep times of wristband-type and self-report had a strong correlation (coefficient value = 0.93, p < 0.001).

**Conclusions:**

This PAT is useful for capturing changes in the amount of physical activity and the daily rhythm within the individual. It can be expected to be used for rehabilitation support centered on upper limb activity and daily rhythm.

## Introduction

Physical activity trackers (PATs) are commonly used to monitor the exertion levels of the elderly and patients in rehabilitation [1] since uncontrolled physical activity levels are crucial in worsening the fatality and secondary morbidities, such as metabolic or locomotive syndromes [2–5]. Physical activity levels also influence the effects of rehabilitation interventions aimed at preventing deterioration in long-term care levels of persons with disabilities along with improving their motivation levels and quality of life (QOL) [5, 6]. Accordingly, PATs extensively used in rehabilitation, so far, were most commonly based on the user’s step counts measured using a pedometer or an accelerometer [7–9].

It is important to index the amount of physical activity and daily rhythm of rehabilitation subjects to formulate an ideal support plan and ensure optimum benefits from the rehabilitation intervention [10, 11]. A PAT is used to collect these indicators accurately and easily. For instance, in patients with chronic obstructive pulmonary disease, a pedometer is often used to monitor daily activity. However, it is noted that user-friendliness (e.g., comfortable to wear, easy to use) is important to keep the device on for a long time [12]. Often, elderly people and patients with dementia have to keep the device on to prevent forgetting to wear it before beginning their physical activity; therefore, it is recommended that a consumer PAT must be easy to apply and durable [13]. In this regard, a waterproof wristband-type PAT is recommended in this population [14, 15]. Additionally, to capture the self-reported daily rhythm, such as sleep time, the subject’s conscious efforts and cognitive abilities are essential for daily recording. Therefore, to mitigate this difficulty in obtaining continuous accurate sleep time recording, a wristband-type PAT is useful.

Step count, which can be measured using a PAT, is a well-known index of physical activity. To improve the accuracy of step count measurement, verification using several research devices has been perfomed [16, 17]. Over the years, several studies have been conducted to evaluate consumer PAT-related ease of use and accessibility. Since 2013, many companies have developed a variety of PATs with multiple studies evaluating their measurement accuracy [18]. Studies on healthy adults using devices worn on various body parts (e.g., chest, waist, hip, and ankle) have revealed that wrist-worn devices were most preferred because of their accuracy in determining the user’s step count [19, 20].

A prominent name in this field is Fitbit, whose devices are often used for their measurement accuracy, and are registered and verified in clinical trials [18]. The measurement accuracy of a wristband-type PAT from Fitbit devices was validated against the measurement accuracy of the gold-standard treadmill, the index of walking movement measured using a waist holder-type PAT, and a self-reported index of energy expenditure and sleep time [21–23]. These studies reported that the step count of the Fitbit device was highly correlated with that of waist holder-type PAT or visual count during treadmill walking. Moreover, consistencies were observed between energy expenditure and sleep time measured using Fitbit devices and the self-reported index, with the error percentage of the predicted value estimated from the device being low level.

However, the wristband-type PAT reportedly overestimated the step count when compared to the waist holder-type PAT under a free-living setting [22]. Some studies stated that the step counts measured by wristband-type PATs were detected during both heavy physical activity, such as wheelchair strokes and arm ergometer revolutions, as well as light upper limb activity, such as playing cards, reading, or writing [24, 25]. A probable reason for this overestimation is that the PAT considers the slightest movement of the upper limbs as a step count. Furthermore, PATs used to capture changes in daily life during fasting (e.g., Ramadan) reported a significant difference in daily activity and sleep time, suggesting the need for support based on daily rhythm such as physical activity and sleeping pattern [26]. Since changes in sleep time due to lifestyle changes must be properly captured and require the patient’s conscious efforts, self-reporting is often difficult.

Therefore, verifying the measurement accuracy of a convenient and comfortable PAT device is essential to ascertain its use for a wide range of patients. Furthermore, if the wristband-type PAT were to purely identify walking, the step count must be 0 in case of isolated upper limb activity. If not, it can be assumed that wristband-type PATs detect some movement of the upper limb as step counts. We identified various conditions measured as step counts by a wristband-type consumer PAT in terms of two elements of upper limb movement, distance and subjective speed, when using upper limbs during daily living. We further examined how elements, such as the magnitude of upper limb activity and movement speed, affect step counts by a wristband-type consumer PAT. Moreover, since lifestyle changes are often reflected in the user’s sleep time, clinically meaningful concordance is expected between the self-reported and wristband-type PAT-estimated sleep time. Accordingly, we evaluated the proportion of agreement between the self-reported sleeping time and the sleeping time estimated from the wristband-type PAT for a certain period as an index of one’s daily rhythm. Using these measures, we aimed to examine the measurement accuracy regarding the amount of physical activity and lifestyle changes based on the upper limb activity measured by a wristband-type PAT.

## Materials and methods

### Participants

Forty participants (20 females, mean age = 32.65 ± 9.52 years) were enrolled in two experiments for this study. The participants were healthy adults aged >20 years without a history of any musculoskeletal, cardiovascular, or respiratory disorder that may restrict their daily physical activity. Those who had difficulty wearing a PAT, i.e., any discomfort or allergic reaction, or could not manage the PAT or answer the questionnaire due to dementia or mental illness, were excluded from the study. None met the exclusion criteria or retired during this study. Data were collected from October 2020 to March 2021.

The study was approved by the Ethics Committee of the Fukuoka International University of Health and Welfare, Fukuoka, Japan (approval number: 20-fiuhw-015). Before beginning the experiments, all participants were informed of their right to participate and withdraw participation during the experiments. Afterward, we obtained written informed consent from all participants.

### Instrumentation and outcomes

#### PAT

**Fig 1:**
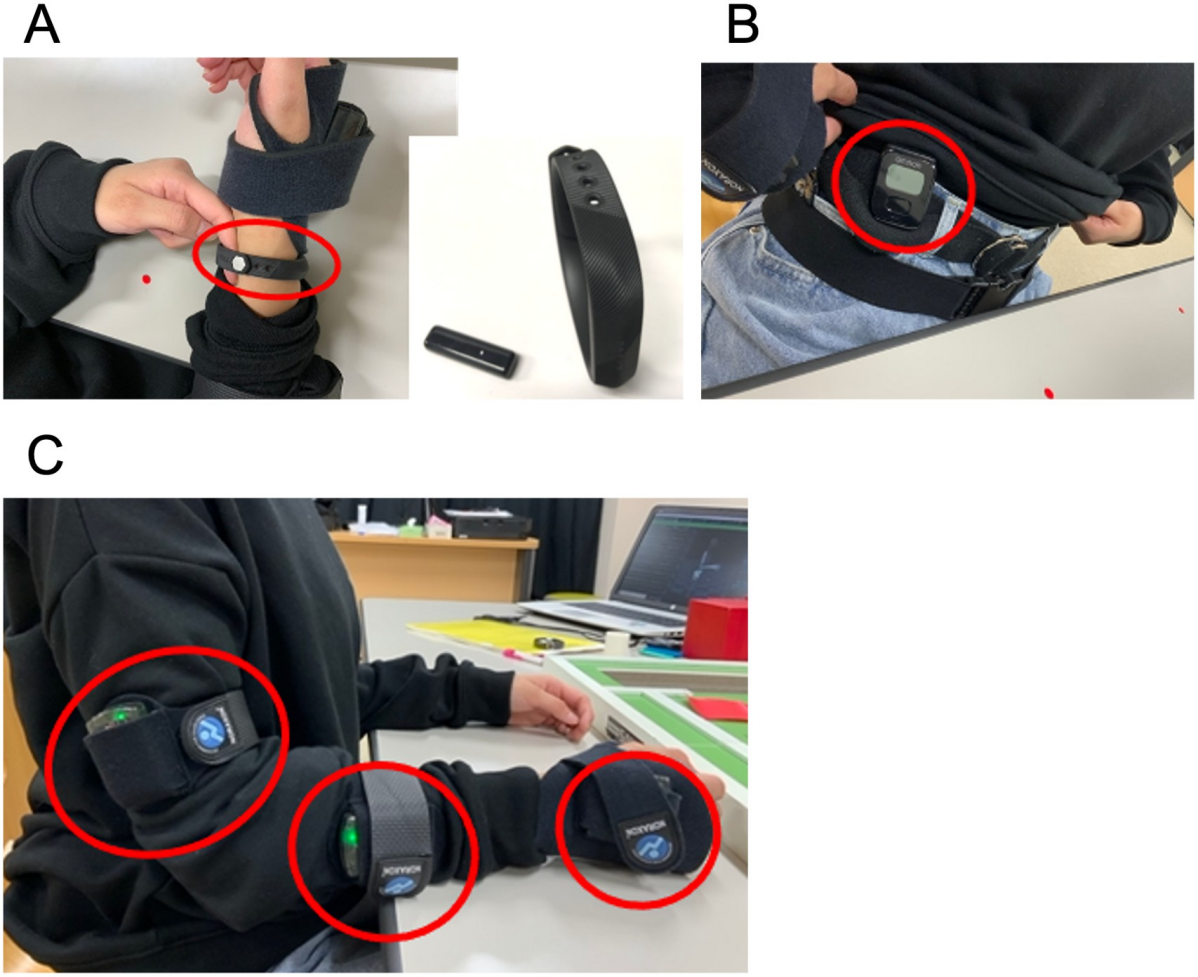
Measuring equipment in this study. A: wristband-type PAT (Fitbit Flex2^™^). B: waist holder-type PAT (Active Style Pro HJA-750C). C: inertial measurement unit sensors of physical motion analysis (MyoMotion).

We chose the Fitbit Flex2^™^ (Fitbit Inc., San Francisco, CA, USA) wristband-type PAT, a tri-axis accelerometer, for this study. The Flex2^™^ has a proprietary algorithm based on wrist movements that captures body movements in a three-dimensional space as the step count, and the time from falling asleep to the time of waking up as the sleep time. It has a Bluetooth 4.0 wireless transceiver, a rechargeable lithium-polymer battery, and is waterproof up to a depth of 50 m (https://www.fitbit.com/is/lookbook/flex2). Data obtained from the Flex2^™^ can be checked via Bluetooth on a tablet or smartphone using the dedicated application (https://www.fitbit.com/global/us/setup). The step count and the sleep time can be measured at any time by synchronizing with the device. Each device used in the study was linked to a single account created on the Fitbit website (https://www.fitbit.com/nl-nl/app#download-now). For research purposes, an anonymous user account was created by our research team and was only accessible to us. The wristband-type PAT was worn around the radial and ulnar styloid processes of the participant’s dominant hand, and a one-finger space (~1 cm) was secured when winding the attached belt (Fig 1A).

Next, we used the Active Style Pro HJA-750C (Omron Healthcare Co., Ltd, Kyoto, Japan) waist holder-type PAT (a tri-axis accelerometer). The device has an algorithm based on waist movements which has been validated [27, 28] to measure the time spent in light physical activity, moderate-to-vigorous physical activity, sedentary behavior, and step counts. The Active Style Pro is often used as an activity tracker for research, and can check the recording of time, step counts, and activity intensity (Metabolic equivalents; METs) by operating the button directly under its display. The recorded data can be extracted using a Bluetooth unit. To ensure that step counts were not measured during upper limb activity, the waist holder-type PAT was worn on the belt loop or top of the pants near the participant’s right anterior superior iliac spine (Fig 1B).

#### Physical motion analysis

We used the MyoMotion (Noraxon Inc., Scottsdale, AZ, USA) system, which is a three-dimensional (3D) inertial measurement unit (IMU) sensor-based motion analysis system, used to measure the movement trajectory of upper limb activities. The IMU sensors include a 3D accelerometer, gyroscope, and magnetometer that measure the 3D rotation angles of each IMU sensor in absolute space. The IMU sensors of MyoMotion can measure data wirelessly and are used in spaces other than the laboratory (https://www.noraxon.com/our-products/3d-motion-capture-imu/).

In this study, to estimate the distance moved by different body parts at the same location as the wristband-type PAT, the position information from the IMU sensor on the dorsal aspect of the dominant hand was recorded and used for analysis (Fig 1C). The measurement conditions in MyoMotion set as the position information of x (latitude), y (meridian), and z (vertical) coordinates of each sensor with respect to the dorsum of both feet (upper foot, slightly below the ankle) were acquired. The position information of the x, y, and z coordinates was obtained using the MR3 software (Noraxon Inc., Scottsdale, AZ, USA) at a sampling frequency at 100 Hz. Distance covered by the movement trajectory (*d*) of the dorsum of the dominant hand during upper limb activity from the position information of x, y, and z coordinates was calculated using the three-square theorem using the following equation:

$$d=\Sigma \sqrt{{\left(x1-x2\right)}^{2}+{\left(y1-y2\right)}^{2}+{\left(z1-z2\right)}^{2}}$$

where, x1, y1, and z1 are the coordinates at the 1/100 second point; x2, y2, and z2 are the coordinates at the 2/100 second point; and Σ represents the sum of the distances between all points within the measured time. Additionally, to check online the motion simulation in the skeletal model for any unusual activity, the remaining IMU sensors were placed according to the following body model: upper thoracic (below the 7^th^ cervical spine in line with the spinal column), pelvic (body area of the sacrum), upper arm of the dominant hand (midway between the shoulder and elbow joints, lateral to the bone axis), forearm of the dominant hand (midway between the elbow and wrist joints, posterior to the bone axis) and the dorsum of the dominant hand.

#### Self-reported sleep state

We asked the participants to report their bedtime and wake-up time during the experiment. As for bedtime, we defined falling asleep as the participants lying down at their usual sleeping place and then closing their eyes, and asked the participants to record just before falling asleep or the time when they seem to have fallen asleep the next morning. We defined the wake-up as the participants getting out of their sleeping place and starting to move, and asked participants to record the time they woke up. We noted the individual sleep times based on the participant-reported bedtime and wake-up time.

#### Upper limb task

**Fig 2:**
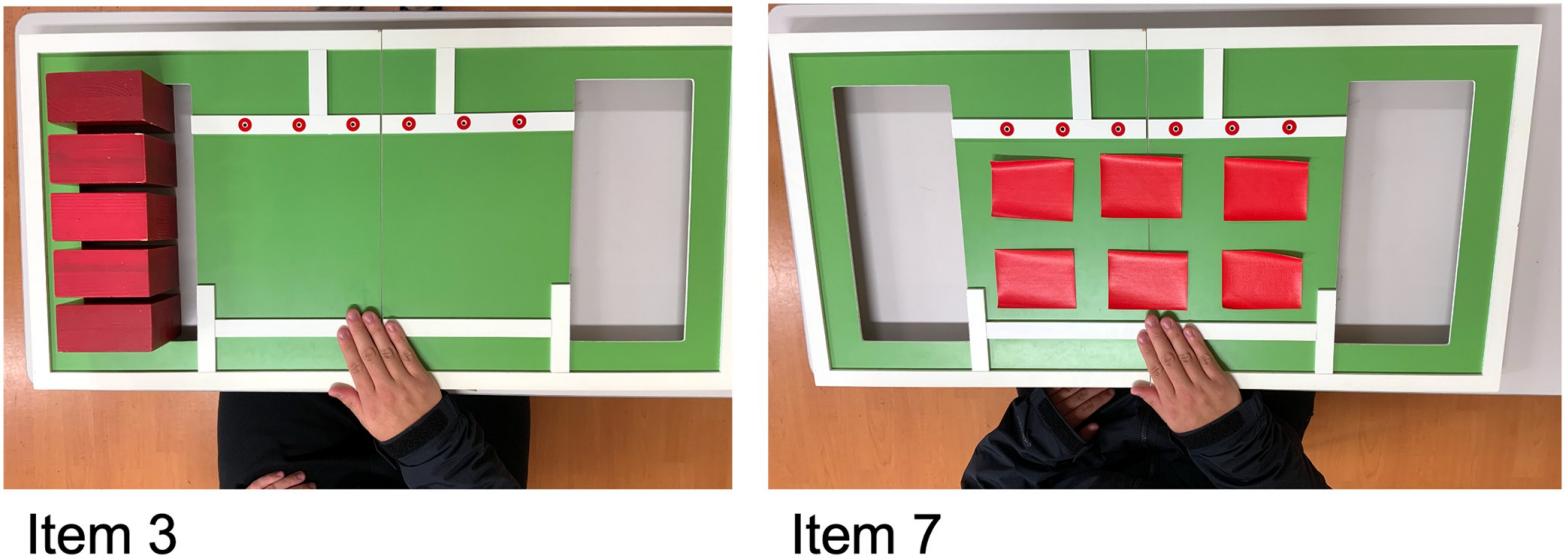
The simple test for evaluating hand function (STEF).

As an upper limb activity task, the participants performed a simple test for evaluating hand function (STEF) that is often used in the evaluation of upper limb function in rehabilitation [29]. Reports have shown that improvement in the upper limb function following rehabilitation intervention was attributed to an improvement in STEF score, and that this improvement in STEF was associated with improvements in activities of daily living expressed as the physical activity of rehabilitation subjects [30, 31]. STEF consists of ten items that include picking up objects, such as balls and pins of various sizes one by one from a storage space and moving them into a target space as quickly as possible. These items are classified into three categories: grip motion (item 1–3), pinch motion (item 4–7), and coordinated motion (item 8–10) [31, 32]. The score of each test in STEF is determined as the time required for picking up and moving; as the tests progress, the objects become smaller and more difficult. For this study, we selected items 3 and 7, which were classified as grip motion and pinch motion, respectively, in STEF. Item 3 is an examination in which the participant grasps and moves five large rectangles (5 cm long, 10 cm wide, and 10 cm tall), and item 7 is requires the participant to pick up six pieces of cloth (9 cm long and 7 cm wide) and turn it over (Fig 2).

### Procedures

#### Experiment 1: Measurement conditions for Flex2 during upper limb activity

With reference to the effect size calculated in a previous study [24], a sample size of 20 participants was recommended based on the effect size f = 0.79, a = 0.05, power = 0.90. A priori power calculation for determining the sample size was performed for a two-tailed test of hypothesis using G*Power 3.1.9. The experimental data were collected from 20 participants (10 males and 10 females, mean age = 27.80 ± 8.73 years, n = 17 right hand dominant). Experiment 1 was conducted to identify the conditions measured as step counts for the wristband-type PAT in terms of two elements of upper limb movement–distance and subjective speed in upper limb activity. To compare the upper limb movement distance, we selected items 3 and 7 in STEF. Item 3 considers that the movement of the upper limbs is the largest, and item 7 considers that it is the least.

To compare the subjective speed in upper limb activity, we defined 10 as the fastest pace and 1 as the slowest pace in the range of speeds at which the participant performs their daily life upper limb activity. Out of 10 levels of subjective activity speed ranging from 1 to 10, the slow condition was to perform at a speed of 20%–30%, and the fast condition meant performing at a speed of 80%–90%. We measured the time taken by the participants to perform the two tasks (item 3 and item 7 of STEF) at these two paces (slow condition and fast condition). The step counts on the wristband-type and waist holder-type PAT were confirmed before and after each trial under the slow and the fast conditions; the step count increase and the time taken for each trial were recorded. We calculated the percentage detected as a step count for each trial. The first two trials of the slow and fast conditions were considered practice trials, and the third trial data was used for analysis.

#### Experiment 2: Consistency with self-reported sleep time

With reference to the effect size calculated in a previous study [33], a sample size of 14 participants was recommended based on the effect size f = 0.70, a = 0.05, power = 0.90. The experimental data were collected from 20 participants (10 males and 10 females, mean age = 37.50 ± 7.76 years, n = 18 right hand dominant). This sample size was chosen since some participations might miss records or withdraw during the measurement span. The wristband-type PAT was worn in the same place as in Experiment 1 and was recovered after a wearing period of 5 days. The data on the self-reported survey were collated with the period during which the wristband-type PAT was worn. The sleep time on the 2^nd^ to 4^th^ days excluding the data from the 1^st^ day when the wristband-type PAT was worn and the data on the 5^th^ day onward were used for the analysis.

### Statistical analyses

**Fig 3:**
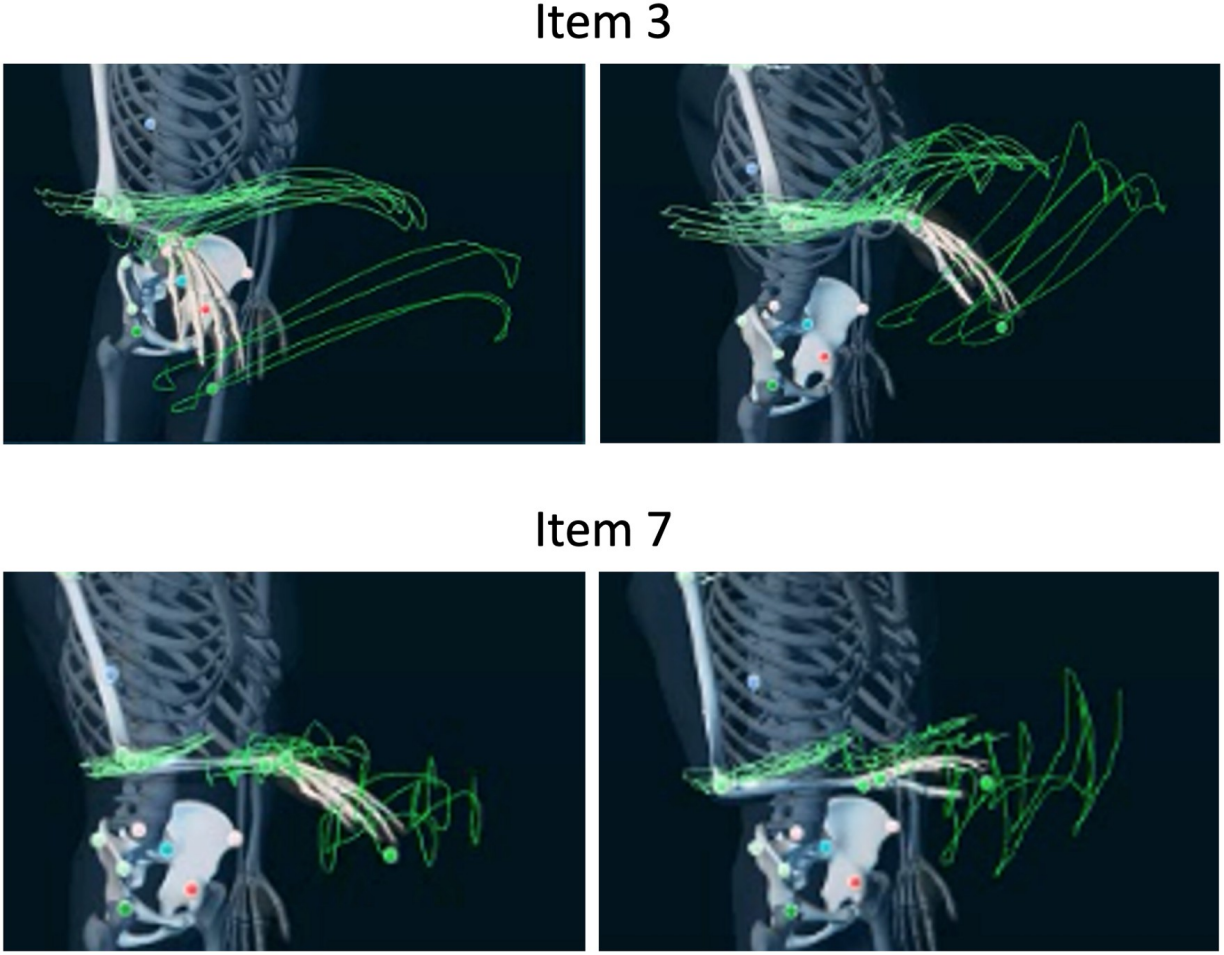
Movement trajectory of upper limb activity during item 3 and item 7 of STEF used by MyoMotion. The left side of the image is the slow condition, and the right side is the fast condition. The green lines in the images show the movement trajectory of the upper limbs.

In Experiment 1, we used a two-way repeated-measures analysis of variance (ANOVA) to confirm whether there is a difference in the distance “*d*” between the two tasks with elements of task (item 3 and item 7) and pace (slow condition and fast condition) (Fig 3). Two-way ANOVA examines how a response variable is affected by two elements, and repeated measures indicates that one of the elements was repeated. Similarly, we used a two-way ANOVA to confirm whether there is a difference in the subjective activity speed in upper limb activity between the two paces with elements of task (item 3 and item 7) and pace (slow condition and fast condition) for the time taken for each trial. To identify the effects of the upper limb movement distance and subjective speed on step counts for PAT, a two-way repeated-measures ANOVA was employed with elements of task (item 3 and item 7) and pace (slow condition and fast condition) for the step counts of wristband-type and waist holder-type PAT.

In Experiment 2, to confirm whether the sleep time captured by the wristband-type PAT demonstrated applicable consistency with the self-reported sleep time and has useful measurement accuracy, Bland–Altman analysis was performed. The Bland–Altman analysis is used to confirm whether there is a systematic error consisting of a fixed error and a proportional error between the two methods [34]. For the sleep time data obtained by self-report (Time1) and the wristband-type PAT (Time2), we verified the fixed error between the two sleep times by drawing a scatter plot of the mean sleep time {(Time1 + Time2)/2} and the error (Time1 − Time2) and calculated the mean error ± 1.96 standard deviations. We also verified the systematic error between the two sleep times by calculating the significant difference in the standardization coefficient (slope) of the regression analysis with error as the dependent variable and the mean sleep time as the independent variable. To verify the relationship between the two sleep times, we calculated the correlation coefficient using Pearson’s correlation analysis between Time1 and Time2. To determine tendencies in the measurement error of the overall sleep time in this study and compare them with those reported the previous study, the mean absolute percentage error (MAPE) was calculated using the following formula:

$$MAPE=\left(\frac{1}{Ν}\Sigma \frac{\left|Time1-Time2\right|}{\left|Time1\right|}\right)\times 100\%$$

All statistical analyses were performed using SPSS version 26 (IBM Inc. Corp., Armonk, NY, USA). In all the analyses, a p-value < 0.05 was considered statistically significant.

## Results

### Experiment 1: Measurement conditions of Flex2 during upper limb activity

**Fig 4:**
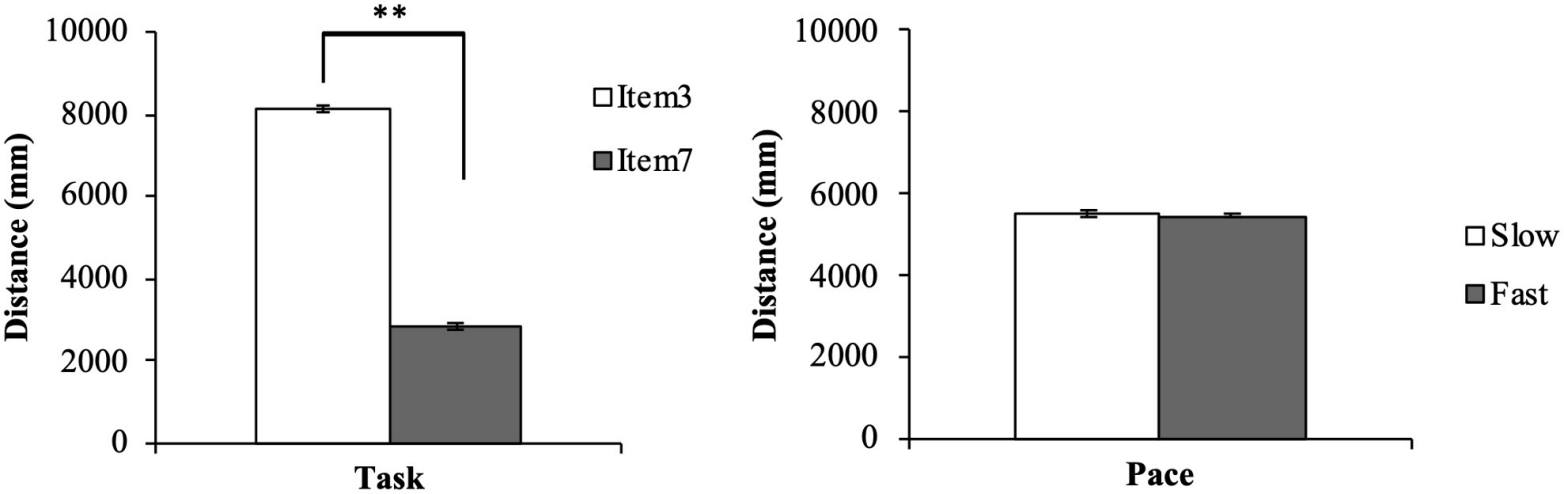
Two-way repeated-measures ANOVA for distance. Distance represents the distance of the upper limb movement, **p < 0.*01*, *p < 0.*05*.

**Fig 5:**
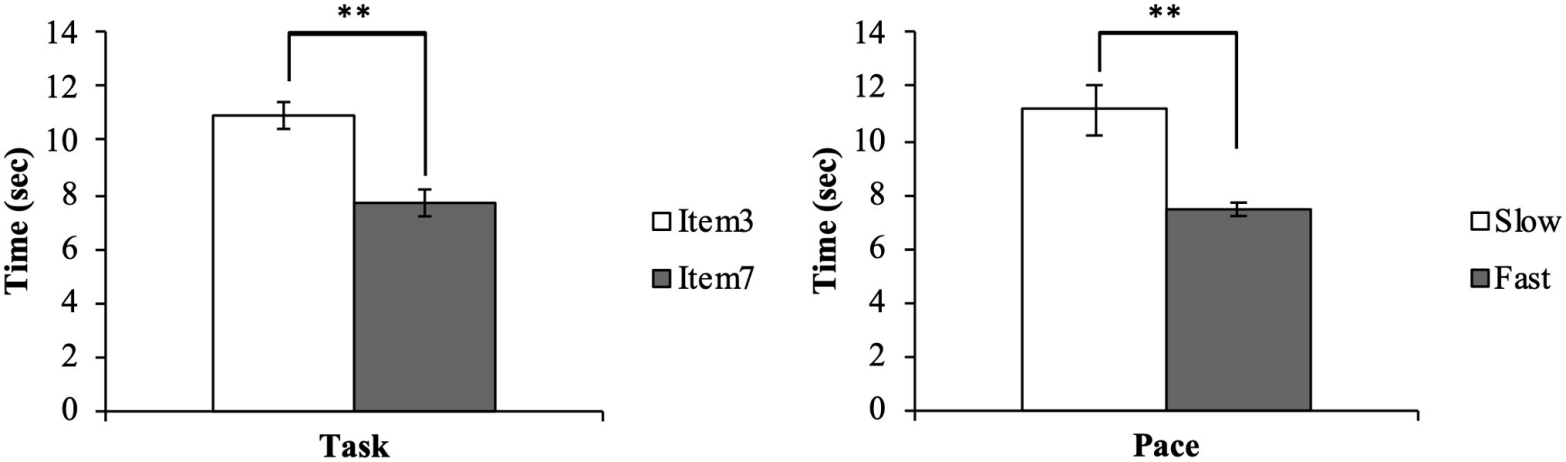
Two-way repeated-measures ANOVA for time. Time represents the time taken for each trial, **p < 0.*01*, *p < 0.*05*.

**Fig 6:**
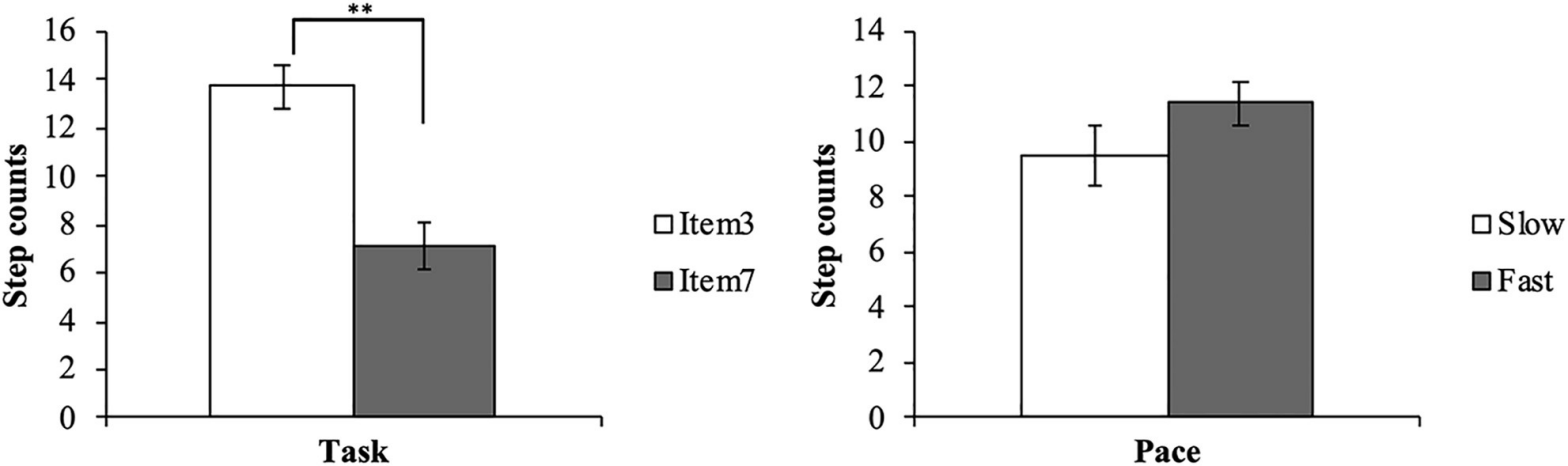
tTask effect for step counts of Flex2. Step counts represent the average step counts measured by Flex2, **p < 0.*01*, *p < 0.*05*.

The average step counts and percentages measured by Flex2, the average distance of the movement trajectory of the dorsum of the dominant hand, and the time taken in each trial (item 3, item 7, slow condition, fast condition) are presented in Table 1. The step counts measured by the waist holder-type PAT were 0 in all trials. Regarding the distance of the movement trajectory, only the task element had the main effect (F (1, 19) = 2629.308, p < 0.001); the distance of the upper limb movement was significantly longer in item 3 than in item 7 (Fig 4). We found no significant main effect for pace (F (1, 19) = 3.411, p < 0.080) and no interaction between task and pace (F (1, 19) = 0.250, p < 0.623). Regarding the time taken for each trial, each element of the task and the conditions had the main effect (F (1, 19) = 162.341, p < 0.001, F (1, 19) = 17.356, p = 0.001, respectively); item 3 took significantly longer time than item 7, and the slow condition took significantly longer than the fast condition (Fig 5). No significant interaction was noted between task and pace (F (1, 19) = 4.035, p < 0.059). As a confirmation of the measurement conditions, it can be interpreted that the distance and time were different between the two items and the time was different between the two subjective speeds. Furthermore, we observed a significant difference only in the main effect of the task, and item 3 was counted significantly more than item 7 (F (1, 19) = 31.705, p < 0.001) (Fig 6). On the other hand, there was no significant main effect for pace (F (1, 19) = 2.669, p < 0.117) and no interaction of the task and pace (F (1, 19) = 1.767, p < 0.200).

**Table 1:** Measurement results of Experiment 1.

		Step counts	Percentage (%)	Distance (mm)	Time (sec)
Item 3	Slow	13.65 ± 7.69	80.00	8165.40 ± 406.98	12.99 ± 4.58
	Fast	13.80 ± 2.84	100.00	8123.51 ± 340.22	8.87 ± 1.36
Item 7	Slow	5.30 ± 5.70	50.00	2870.27 ± 352.53	9.26 ± 3.51
	Fast	9.00 ± 5.66	75.00	2790.31 ± 308.39	6.10 ± 1.25

### Experiment 2: Consistency with self-reported sleep time

**Fig 7:**
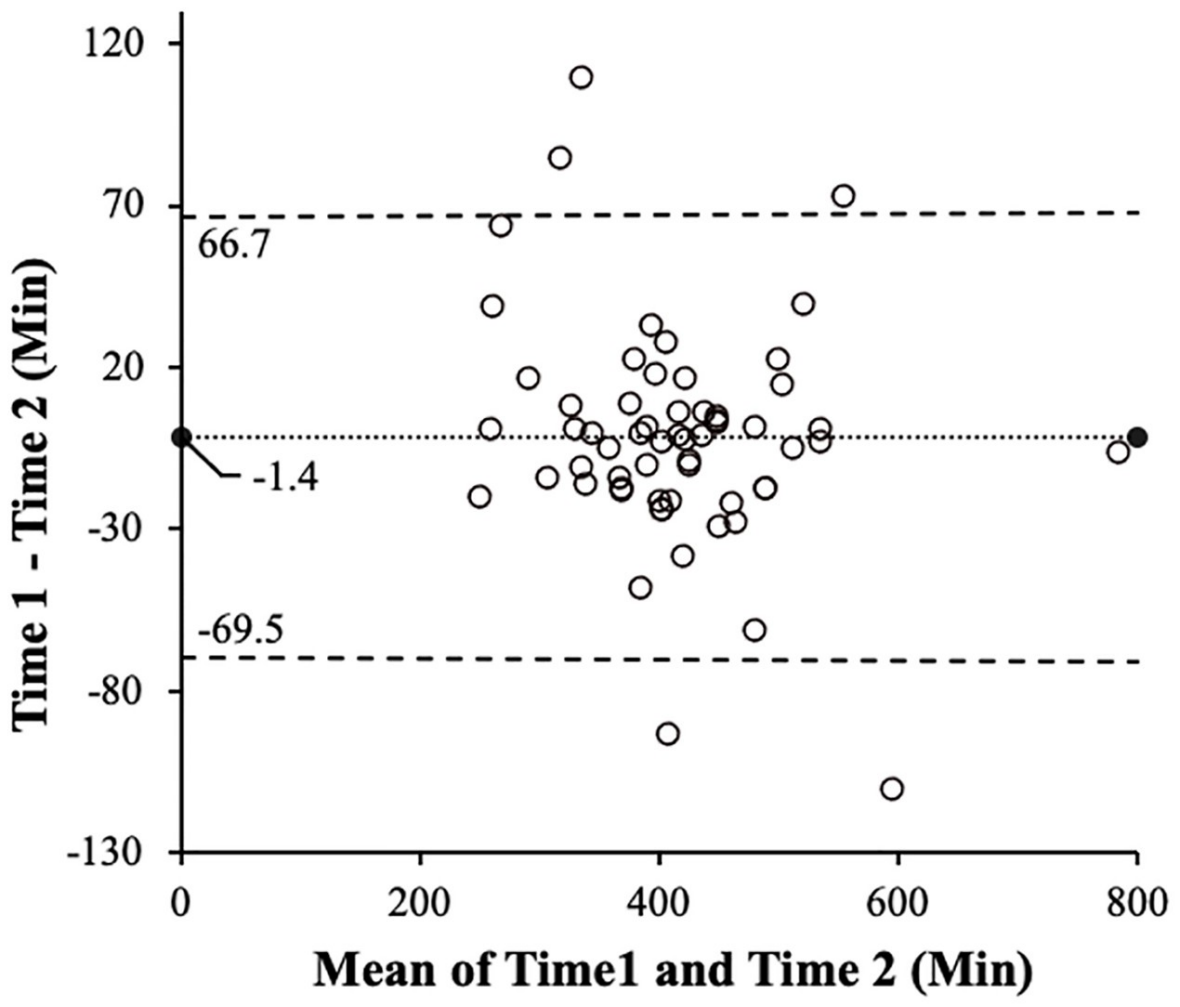
Scatter plot of the mean and error of two sleep times. The finer dashed line represents the mean error, and the larger dashed line represents the range of the mean error ± 1.96 standard deviation.

**Fig 8:**
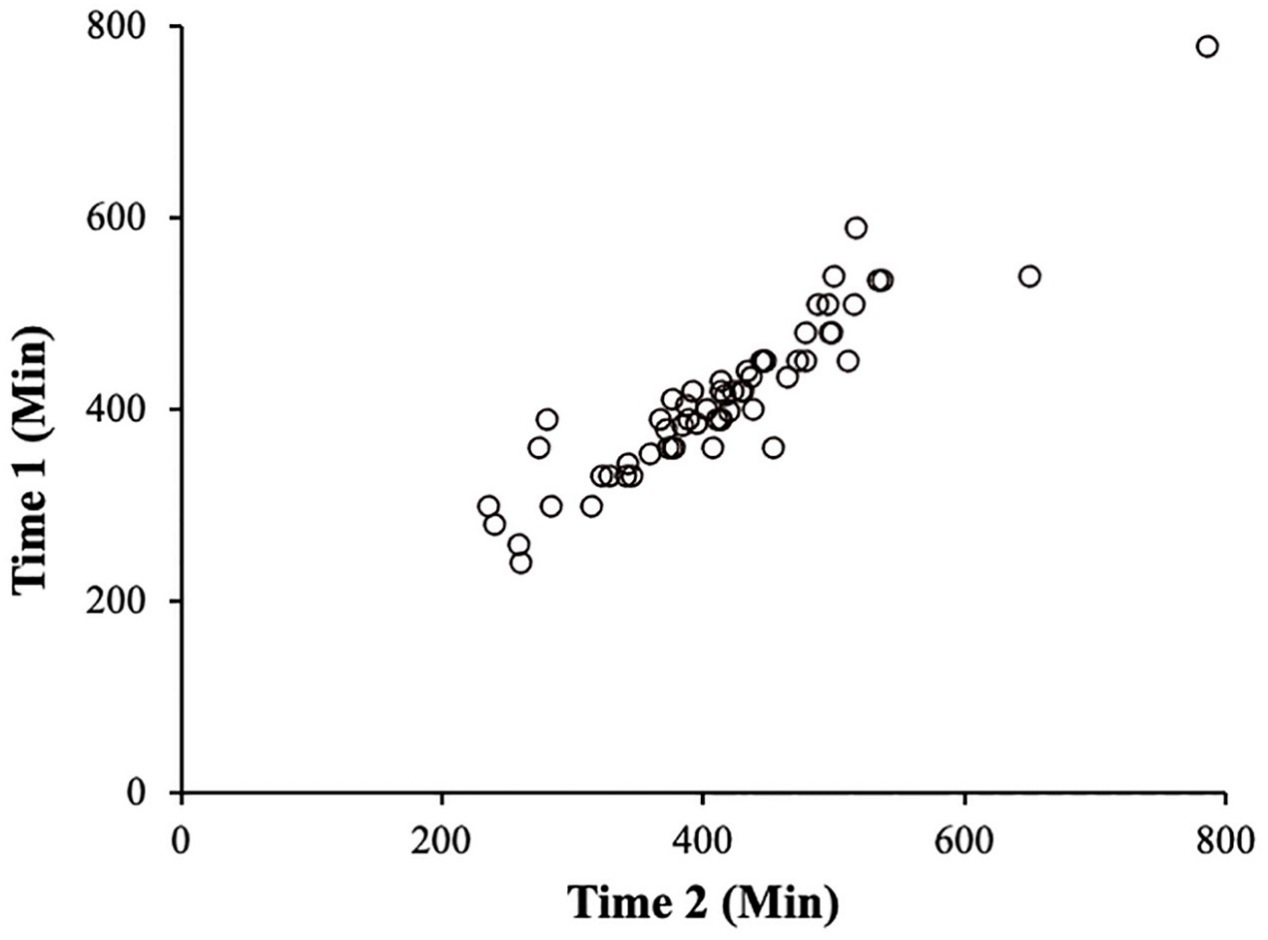
Scatter plot using Pearson’s correlation analysis.

None of the participants removed the wristband-type PAT or withdrew from the experiment. The average sleep time of Time1 was 413.43 ± 87.21 minutes, while that of Time2 was 414.85 ± 94.62 minutes. The Brand Altman analysis revealed that the mean error of sleep time (Time1 − Time2) was −1.42 ± 34.74 minutes, and the mean error ± 1.96SD (−69.50–66.67) crossed 0, so there was no fixed error (Fig 7). In the regression analysis, the intercept measured 33.56 (95% confidence intervals (CI): −8.70–75.82, p = 0.12) and the slope was −0.08 (95% CI: −0.18–0.02, p = 0.10), so there was no systematic error. Lastly, Pearson’s correlation coefficient value was 0.93 (95% CI: 0.89–0.96, p < 0.001), showing a very strong correlation (Fig 8). The MAPE in sleep time data obtained by the self-report and wristband-type PAT was 5.65%. Since the range of MAPE has been reported to be 4–20% in previous studies [22, 33], the MAPE obtained in the current study can be considered low.

## Discussion

This study examined the measurement accuracy of the wristband-type PAT regarding the amount of activity with the upper limbs and capturing the sleep time as the daily rhythm. We found that the wristband-type consumer PAT measured the step counts even when performing upper limb activity with the dominant hand. We also observed that measuring the upper limb activity as step counts were not affected by the subjective speed of movement, but rather by upper limb movement distance. In addition, the sleep time estimated by the wristband-type PAT was considerably consistent with the self-reported sleep time, and the wristband-type consumer PAT can effectively capture the daily rhythm in free-living life.

### Measurement conditions of Flex2 during upper limb activity

As per the study hypothesis, the waist holder-type PAT did not detect step counts with upper limb activity in all the trials of experiment 1, but they were significantly detected by the wristband-type PAT. Therefore, it can be inferred that the wristband-type PAT overestimated the physical activity because the upper limb movement was measured in addition to the movement measured during walking in a free-living setting other than the experimental setting (e.g., walking on the treadmill, trial of a fixed number of steps).

In previous studies, the measured step counts differed depending on certain factors, such as the type and speed of upper limb activity [24, 25], so we considered that the two elements of movement distance and subjective speed of upper limb activity were involved. Accordingly, we examined these two elements and identified that only the upper limb movement distance affected the step count accuracy as estimated by the wristband-type PAT. As an element of the movement distance of the upper limbs, the wrist-band type PAT identified the upper limb activity as step count whenever the hand moved greatly, such as in moving the object from one side to the opposite side in item 3, but a small hand movement, such as turning over the cloth in item 7, was insufficient to be identified as a step count. The two items of STEF used in the current study can be classified into a grip motion composed of grasping objects and coarse transportation and a pinch motion composed of the manipulation using the finger pulp [31, 32]. The difference in distance between the two items classified as different motions in STEF could be clearly verified. Moreover, our finding showed that the difference in upper limb activity is important for the PAT to detect changes in physical activity. Notably, the subjective speed in upper limb activity was not involved in the step count. Since the algorithm of PAT is not publicly available, it is reasonable to assume that step counts are detected based on the normal walking algorithm; therefore, the results detected by upper limb activity may change as the subjective speed of upper limb activity increases or decreases. It has been reported that in the walking step count measurement, a measurement error occurs depending on the walking stroke, walking speed, and walking state (e.g., using a cane, walker) with respect to the step counts measured in the experimental setting [22, 35, 36]. It is difficult to achieve absolute measurement accuracy even in regular walking movements; hence, it is necessary to accurately comprehend the characteristics and precautions of the device before using it as an objective index. Thus, the characteristics of upper limb activity measured as step counts are synonymous with characteristics that are identified as the step counts of walking using PAT. The results of this study offer significant insights into the characteristics of upper limb activity as captured by the wristband-type PAT.

### Consistency with self-reported sleep time

As a characteristic of the wristband-type PAT, its usefulness as a device to capture changes in the user’s lifestyle was examined. A comparison with a wristband-type PAT based on polysomnography and systematic reviews reported the relationship with sleep time [18, 21, 22]. Fitbit devices are one of the most popular consumer wearable PATs, and although the comparison with the research-standard criterion shows the possibility of overestimation or underestimation, a significant positive relationship with the standard measurement method has been reported.

In this study, we verified the proportion of agreement between the self-reported sleep time and the sleep time estimated from the wristband-type PAT as an indicator of one’s daily rhythm. We found that the sleep time obtained from the two measuring methods was a comparable value and that homogeneous measurement accuracy was secured. Since no systematic error occurred, it can be interpreted that the errors between the two sleep times are caused by chance, rather than the errors in the estimated sleep times due to specific causes, such as the accuracy of the device and the evaluation method. Therefore, it can be inferred that the sleep time obtained by using the wristband-type PAT reflects the self-reported sleep time and that the time of wakefulness other than sleep can be measured almost accurately. The MAPE calculated to determine the overall tendency in the measurement error showed a low level of error tendency even when compared with previous studies [33]. This indicates that the error in sleep time estimated from the device tends not to deviate significantly.

The wristband-type PAT used in this study was waterproof and had an active time of about 5 days after fully charged. The device was selected since it can detect reliable sleep time data for at least 3 days without any special operation. Since no display presents the current step count and activity intensity, there was no concern about information bias to the participants. Our results also suggest that, unlike the self-reported method, the wearable PAT can be expected to measure the individual’s life rhythm from their sleep and wakefulness patterns without the need for conscious efforts for daily recording and sufficient cognitive ability.

### Clinical implications

Previous studies have reported that consumer PATs, such as those manufactured by Fitbit, Garmin and Jawbone are worn by rehabilitation subjects to capture the outcome of physical activity [13, 23, 37, 38]. However, since these devices use step counts and estimated energy expenditure as the main outcomes, one could conjecture that the physical activity consists only of walking. Therefore, the influence of step count detected by consumer PATs due to upper limb activity is not considered, and limited studies have been available on the introduction of activity tracker in people who need equipment or assistance for walking, including wheelchair users.

Previous studies have shown that the wristband-type consumer PATs are not practical for comparative assessment with others, but that they can be used for capturing changes within the user [38–40]. It is known that if appropriate support is not provided to the patient population undergoing rehabilitation, mortality and the degree of long-term care may worsen due to a decrease in physical activity and disrupted sleep cycle, leading to irregular daytime sleeping [3, 4, 41]. In addition, using a device that needs to be attached/detached or charged during the day (e.g., waist-type or cumbersome size) may be difficult due to deteriorated physical function, or the risk of forgetting to wear or losing it due to cognitive problems [13].

The wristband-type PAT offers a feasible solution to capture these situations more easily and accurately, which allows for early countermeasure and verification of individual state changes obtained by appropriate intervention. Regarding measuring the physical activity of the patient population using the wristband-type PAT, it is necessary to consider the characteristics of upper limb activity, which are easily measured as steps counts. Nevertheless, the device is expected to accurately capture the within-subject changes in the amount of physical activity and the daily rhythm. The wristband-type PAT used in this study can be used for capturing changes in patients who have difficulty walking, and for behavioral approaches centered on upper limb activities, such as self-care and instrumental activities of daily living (e.g., domesticities, hobbies) in rehabilitation intervention.

### Limitations

There were some limitations to this study. First, a repetitive movement was set as the task, so the effect of free movement was not sufficiently examined. To capture the movements of normal daily life as step count, the movements by upper limb activity and walking, as well as the movements by other unknown factors may be included as step counts. Therefore, even if the amount of activity undertaken by the device user increases, it is not clear how much it is reflected as step counts by wristband-type PAT. Second, participants were healthy adults; therefore, the results cannot be generalized to rehabilitation patients. Nevertheless, we believe that our findings may form the basis of clinical application because devices for which basic accuracy and safety have not been ensured cannot be used experimentally in clinical practice. Finally, in the verification of sleep time in Experiment 2, the participant’s occupations, how they spend their holidays, and how this affected the measurement of sleep time were not investigated. Further studies need to be developed to identify factors, such as occupational and hobby effects, and predictor effects on a regular sleep and daily rhythm in rehabilitated patients.

## Supporting information

S1 Dataset (XLSX)
